# Minocycline and Fluconazole Have a Synergistic Effect Against *Cryptococcus neoformans* Both *in vitro* and *in vivo*

**DOI:** 10.3389/fmicb.2020.00836

**Published:** 2020-05-05

**Authors:** Qinxiang Kong, Zubai Cao, Na Lv, Hui Zhang, Yanyan Liu, Lifen Hu, Jiabin Li

**Affiliations:** ^1^Department of Infectious Diseases, The First Affiliated Hospital of Anhui Medical University, Hefei, China; ^2^Department of Infectious Diseases, Chaohu Hospital of Anhui Medical University, Hefei, China; ^3^Anhui Center for Surveillance of Bacterial Resistance, Hefei, China; ^4^Institute of Bacterium Resistance, Anhui Medical University, Hefei, China

**Keywords:** minocycline, fluconazole, synergism, *Cryptococcus neoformans*, *Galleria mellonella* model

## Abstract

In recent decades, the incidence of *Cryptococcus neoformans* infection, which causes cryptococcosis, has consistently increased. Fluconazole (FLU) is frequently used in the treatment of this disease, mainly in the immunocompromised population, and long-term therapy usually produces drug resistance. Research on antifungal sensitizers has gained attention as a possible means of overcoming this drug resistance. Minocycline (MINO) has an inhibitory effect *in vitro* on FLU-resistant *Candida albicans*, and the combination of MINO and FLU has a synergistic effect on FLU-resistant *C. albicans*. A synergistic effect of MINO/FLU has been reported against *C. neoformans*, but this effect has not been evaluated on FLU-resistant isolates. This study aimed to investigate the interaction of MINO and FLU against FLU-resistant *C. neoformans* both *in vitro* and *in vivo*. We found that the combination of MINO and FLU had a synergistic effect on FLU-resistant *C. neoformans in vitro*. For all FLU-resistant strains, the minimum inhibitory concentration (MIC) of FLU decreased significantly when used in combination with MINO, dropping from >128 μg/ml down to 4–8 μg/ml. Additionally, MINO and FLU had a synergistic effect on both susceptible and resistant *C. neoformans* biofilms, in which the MIC of FLU decreased from >256 μg/ml down to 4–16 μg/ml. Compared with FLU alone, the combination of MINO with FLU prolonged the survival rate of *Galleria mellonella* larvae infected with FLU-resistant *C. neoformans*, and also significantly decreased the fungal burden of infected larvae and reduced the tissue damage and destruction caused by FLU-resistant *C. neoformans*. These findings will contribute to the discovery of antifungal agents and may yield a new approach for the treatment of cryptococcosis caused by FLU-resistant *C. neoformans*.

## Introduction

Invasive fungal infections have become a significant cause of morbidity and mortality in recent years (Brown et al., 2012). *Cryptococcus neoformans* is one of the most common infectious pathogens in immunocompromised individuals, causing life-threatening pneumonia and meningoencephalitis (Recio and Perez-Ayala, 2018). The number of *C. neoformans* cases has increased exponentially in the last 30 years due to the advent of AIDS, the use of immunosuppressive therapy in transplant patients, and the use of chemotherapeutic agents (May et al., 2016).

In clinical practice, fluconazole (FLU) is the most commonly used drug for the treatment and prevention of *C. neoformans* infection, and FLU has been used as the recommended treatment for many years (Williamson et al., 2017; Schwartz et al., 2018). However, the broad utilization of FLU has led to the rapid emergence of drug-resistant isolates (May et al., 2016). Among 4,995 clinical strains isolated from 3,210 patients, the FLU resistance rates were found to be 10.6% in first-time cases and 24.1% in patients with recurrent infection (Bongomin et al., 2018). Therefore, there is an urgent need to develop new alternative drugs for treating *C. neoformans* infection.

Minocycline (MINO), a derivative of tetracycline, is a broad-spectrum antimicrobial agent that inhibits bacterial protein synthesis. It is fat-soluble and can quickly enter the central nervous system through the blood–brain barrier; it also has a broad spectrum of antibacterial activity against both aerobic and anaerobic Gram-positive and Gram-negative microorganisms (Garrido-Mesa et al., 2013; Adibhesami et al., 2015). MINO has been reported to have an antifungal effect when used alone or in combination with other antimicrobial drugs (Jesus et al., 2016; Gu et al., 2018). Furthermore, MINO was found to work synergistically with FLU against clinical isolates of *Candida albicans* and *C. neoformans* (Shi et al., 2010; Gu et al., 2018). Notably, prior studies were conducted on drug-susceptible strains, so there is a limited understanding of the effectiveness of this combination (MINO/FLU) against FLU-resistant *C. neoformans in vitro* as well as a lack of demonstration of their synergy in an *in vivo* model.

A biofilm is a microbial community on a solid surface attached to an external polymer matrix. *C. neoformans* biofilms consist of a complex network of yeast cells fused with a large amount of polysaccharide matrix (Kumari et al., 2017). It has been reported that *C. neoformans* can form biofilms in the drainage tubes of ventricular shunts (Mayer and Kronstad, 2017). Previous studies have reported that biofilms play a role in antimicrobial resistance in *C. albicans* (da Silva et al., 2016). We hypothesized that MINO/FLU could exert an antimicrobial effect against FLU-resistant *C. neoformans* via inhibiting biofilm formation. We were unable to find any prior studies on the combination of MINO and FLU against FLU-resistant *C. neoformans* and biofilms. Therefore, in the present study we systematically evaluated this, both *in vitro* and *in vivo*.

*Galleria mellonella* is a species of wax moth. A model system using the caterpillar stage of this moth has many advantages over traditional mammalian models. First, the larvae have both cellular and humoral defenses, including the production of antimicrobial peptides, which is similar to the innate immune response of mammals. Second, the insects can be infected by injection without anesthesia and maintained at 37°C. Finally, a *G. mellonella* model is not subject to the ethical restrictions of mammalian models. These factors make *G. mellonella* an ideal preliminary infection model. Based on our successful application of this model to verify bacterial infection in previous studies, we used it for our *in vivo* experiments in the present work as well (Li et al., 2018; Lu et al., 2018; Trevijano-Contador and Zaragoza, 2018).

To test our hypothesis, we conducted an evaluation of the *in vitro* antifungal activity of MINO alone or combination with FLU and used a reduction assay of 2,3-Bis-(2-methoxy-4-nitro-5-sulfophenyl)-2H-tetrazolium-5-carboxanilide (XTT) to determine the antibiofilm effects of MINO combined with FLU. Confocal laser scanning microscopy (CLSM) was used to assess the biofilm activity. Furthermore, the interactions of drug combinations *in vivo* were evaluated by establishing a *G. mellonella* infection model and assaying the impact of MINO and FLU used alone and in conjunction on the survival rate and fungal burden. Histological sections of the larvae were examined as well.

## Materials and Methods

### Strains, Media, and Antimicrobials

The strains used in this study are listed in Table 1. *C. neoformans* strains CN1, CN2, CN4, and CN7 were isolated from the cerebrospinal fluid of hospitalized patients who were diagnosed with cryptococcal meningitis at a tertiary-care hospital located in Anhui, China. *Candida parapsilosis* ATCC 22019, *Candida krusei* ATCC6258 (NIH, Bethesda, MD, United States), and H99 (ATCC208821, NIH) were used as quality controls.

**TABLE 1:** *In vitro* interactions of MINO with FLU against *C. neoformans*.

Strains^a^	MIC_80_ (μ g/ml)^b^				FICI^b^	IN^c^
	Alone		Combined			
	FLU	MINO	FLU	MINO		
CN1	1	>128	0.5	32	0.75	ND
CN2	2	>128	0.5	64	0.75	ND
CN4	4	>128	2	32	0.75	ND
CN7	4	>128	2	64	1	ND
H99	4	>128	2	64	1	ND
CN18	>32	>128	4	32	0.38	SYN
CN26	>32	>128	8	8	0.31	SYN
CN45	>32	>128	8	16	0.38	SYN
CN117	>64	>128	4	16	0.19	SYN
CN225	>64	>128	4	8	0.13	SYN
CN436	>64	>128	8	16	0.25	SYN
CN526	>128	>128	4	8	0.09	SYN
CN593	>128	>128	4	8	0.09	SYN
CN641	>128	>128	8	8	0.13	SYN

*^a^CN, Cryptococcus neoformans; ^b^MIC_80_, the minimum inhibitory concentration of drug that inhibited fungal growth by 80% compared with the growth control; FICI, fractional inhibitory concentration index; MIC_80_ values and FICIs are the median of three independent experiments; and ^c^IN, interpretation; SYN, synergism; ND, no difference.*

This study was carried out in accordance with the recommendations of international ethical guidelines for biomedical research involving human subjects (CIOMS), and the protocol was approved by the Committee on Medical Ethics of The First Affiliated Hospital of Anhui Medical University. All subjects gave written informed consent in accordance with the Declaration of Helsinki.

Yeasts were maintained on Sabouraud dextrose agar (SDA) or L-glutamine-containing RPMI-1640 medium (Sigma-Aldrich, St. Louis, MO, United States) without sodium bicarbonate, which was then supplemented with 2% glucose and buffered to pH 7.0 using 0.165 M MOPS (Sigma-Aldrich). For the *in vivo* experiment, *G. mellonella* larvae (Kaide Ruixin Co., Ltd., Tianjin, China) that were all similar in weight (ca.0.25 g) and absent of gray markings were selected.

All antibiotics were obtained from Sigma-Aldrich China, Inc. Minimum inhibitory concentrations (MICs) were determined as specified by the Clinical and Laboratory Standards Institute standard M27-A3 document (Clinical and Laboratory Standards Institute, 2008), with modifications as described previously (Movahed et al., 2016). Following the manufacturer’s instructions, stock solutions of FLU and MINO were dissolved with dimethyl sulfoxide (DMSO), and ampicillin was prepared in sterile distilled water. Stock solutions were all sterilized using 0.22-μm microfilters, aliquoted, and stored at −20°C until use.

### Construction of the FLU-Resistant *C. neoformans* Strains

The FLU-resistant *C. neoformans* strains were constructed as described previously (Silva et al., 2011). Briefly, the clinical isolates were inoculated on SDA plates to recover their vitality. Each monoclonal strain was inoculated in 10 ml of RPMI-1640 and cultured in flasks shaken at 180 r/min at 35°C for 3 days, after which the concentration was adjusted to 10^8^ colony-forming units (CFU)/ml. Each strain (10 μl per strain) was inoculated into 10 ml of RPMI-1640 with FLU (2 × MIC), and 1 ml of each fungal suspension was then frozen at -80°C. The concentration of FLU (4 × MIC, 8 × MIC, 16 × MIC, 32 × MIC, 64 × MIC) was gradually increased using the above methods until the target concentration of 64 × MIC was reached. Fungal suspensions of the mutant colonies were made and starved for 4 h, after which the suspensions were used to inoculate RPMI-1640 containing different concentrations of FLU to test the genetic stability of the mutant colonies. The FLU-resistant strains selected for further use (CN18, CN26, CN45, CN117, CN225, CN436, CN526, CN593, and CN641) are described in Table 1.

### Antifungal Activities of MINO and FLU

The antifungal activities of drugs against *C. neoformans* were determined using the broth microdilution method in accordance with the Clinical and Laboratory Standards Institute standard M27-A3 document. The test was performed in 96-well microtiter plates on yeast at a concentration of 2.5 × 10^3^ CFU/ml in RPMI-1640 medium (pH 7.0) buffered with MOPS. The final drug concentrations in the wells ranged from 1–256 μg/ml for MINO and 0.25–256 μg/ml for FLU, RPMI-1640 medium was used as a negative control, and a drug-free well was set as the growth control. After 72 h of incubation at 35°C, the MICs were determined by both visual reading and measurement of the optical density (OD) with a microplate reader at 562 nm (Movahed et al., 2016). The MICs were defined as the lowest concentration of drug that still enabled 80% of fungal growth inhibition compared with the drug-free control.

### Synergy Testing by the Checkerboard Method

The synergy between FLU and MINO was assessed by the checkerboard broth microdilution method, as described previously (Lu et al., 2018). In brief, 96-well microtiter plates were set up with increasing concentrations of FLU (0.25–128 mg/L) in the horizontal wells and of MINO (1–128 mg/L) in the vertical wells. Samples of fungal cell suspension were subsequently added to each well at a final concentration of 10^5^ CFU/ml. Plates were incubated for 72 h at 37°C, and the growth in each well was quantified by both visual observation and use of a microplate reader in the same manner as described for the susceptibility testing.

To compare the intensity of the drug interactions, the obtained information was analyzed by two models: the fractional inhibitory concentration index (FICI) model based on the Loewe additivity principle and the Δ*E* model based totally on the Bliss independence (BI) concept. The FICI was calculated using the following equation: FICI = FIC_A_ + FIC_B_ = [MIC_(A–combo)_/MIC_(A–alone)_] + [MIC_(B–combo)_/MIC_(B–alone)_] and interpreted as synergistic when FICI was ≤0.5, antagonistic when FICI was >4, and indifferent when FICI was between 0.5 and 4. The following equation was used to describe the ΔE model: Δ*E* = E_A_ × E_B_ - E_measured_, where E_A_ and E_B_ are the experimental percentages of growth when drugs act alone, and E_measured_ is the measured percentage of growth with the theoretical combination of drugs A and B. When the mean Δ*E*, as well as its 95% confidence interval, was positive, it was taken to indicate a significant degree of synergy. When the mean ΔE, as well as its 95% confidence interval, was negative, it was taken to indicate a significant level of antagonism. In any other case, the conclusion was Bliss’ independence. To summarize the entire interaction, the sum percentages of all significant synergistic (ΣSYN) or antagonistic (ΣANT) interactions were calculated. Interactions with a sum percentage of ≥200% were considered strong, those with a value of 100–200% were considered moderate, and those with a value of <100% were considered weak.

### Time-Kill Assays

Time-kill assays were conducted for each strain using FLU alone or in combination with MINO in accordance with previously described methodology (Sangalli-Leite et al., 2016; Xing et al., 2019). H99 cells (2.5 × 10^3^ cells/well) were treated with 4 mg/L FLU, 128 mg/L MINO, or a combination of 2 mg/L FLU and 64 mg/L MINO. CN526 cells (2.5 × 10^3^ cells/well) were treated with 128 mg/L FLU, 128 mg/L MINO, or a combination of 4 mg/L FLU and 8 mg/L MINO. The suspensions were inoculated on SDA supplemented with 100 mg/ml ampicillin and were incubated at 37°C for 72 h. The number of CFU was determined by estimating the decrease in viable cell number after different lengths of time in contact with the various treatments.

### Biofilm Production, Metabolic Activity, and Anti-biofilm Activity Testing

#### Biofilm Production

Biofilm production of the isolates used in this study was evaluated using a previously described method with some modifications (Lu et al., 2018; Tavares et al., 2019). Briefly, a suspension was generated and adjusted to a concentration of 2.5 × 10^7^ CFU/ml. Then, 20 μl of this suspension was placed within the well of a 96-well microtitration plate, and 180 μl of RPMI-1640 supplemented with glucose (final concentration 8%) was added. Plates were incubated at 37°C for 24 h without agitation. After incubation, the wells were washed twice with sterile phosphate-buffered saline (PBS), and their OD values were determined at 493 nm using a microtitration plate reader. The obtained OD values were used to calculate percent transmittance (%T) values. Through assessing the antifungal activities of MINO/FLU and conducting synergy testing by the checkerboard method, we identified CN526 as a drug-resistant strain. Later, in the biofilm production experiment, we found that CN526 was a high-biofilm-producing strain. Based on these findings, we selected CN526 for further use as a representative high-biofilm-producing strain with which to study the role of MINO/FLU *in vivo* and *in vitro*.

#### Biofilm Metabolic Activity

The metabolic activity of the biofilms was evaluated based on semi-quantitative measurements of planktonic cells (Benaducci et al., 2016; Tavares et al., 2019) and a reduction assay of 2,3-Bis-(2-methoxy-4-nitro-5-sulfophenyl)-2H-tetrazolium-5-carboxanilide (XTT). The metabolic activity of the cells was estimated based on a measurement of their mitochondrial dehydrogenase activity, which reduces the XTT tetrazolium salt to formazan salt, resulting in a colorimetric change. For this assay, 50 μl of XTT solution (1 mg/ml in PBS) and 4 μl of a solution of menadione were added to each well. The microplates were incubated at 37°C and measured at five intervals (2, 5, 8, 24, 48, and 72 h) at 493 nm.

#### Anti-biofilm Activity Testing

Three high biofilm-producing strains (CN18, CN117, and CN526) and the H99 strain were used to test the interactions of MINO with FLU against pre-formed biofilms. Briefly, 200 μl of *C. neoformans* (2.5 × 10^7^ CFU/ml) were added to 96-well microtiter plates and cultured at 37°C for five lengths of time (4, 8, 12, 24, and 36 h) to pre-form biofilms at different maturation stages. The biofilms were then washed three times with sterile PBS, and drugs were added to the biofilm-coated wells at final concentrations of 16–256 μg/ml for MINO and 4–256 μg/ml for FLU. After incubation at 37°C for 24 h, the XTT reduction assay was performed to detect the metabolic activity of the biofilms. Colorimetric changes resulting from the XTT reduction were measured with a microplate reader at 493 nm. The sMIC was defined as the lowest concentration of drug that inhibits the metabolic activity of the biofilm by 80% compared with the drug-free control. In all experiments, RPMI-1640 was included as a negative control.

### Confocal Laser Scanning Microscopy (CLSM)

Pre-formed 24-h biofilm formations, generated as described above, were incubated for 45 min at 37°C in a solution of 25 μg/ml CAAF 488 (Concanavalin A conjugated to Alexa Fluor 488, CAAF; Molecular Probes, Inc. PO Box 22010, Eugene, United States) and 10 μg/mL FUN 1 (Molecular Probes) (Martinez and Casadevall, 2005) generated by combining 4 μl of FUN 1 (10 mM) and 15 μl of CAAF 488 (5 mg/ml) with 3 ml of sterile PBS (Martinez and Casadevall, 2005; Tavares et al., 2019). Subsequently, the coverslips were washed with distilled water taken from the wells and inverted over 4 μl of Fluoromount-G (Sigma-Aldrich, United States) that was previously deposited on microscope slides for observation under a confocal microscope (LSM 510 META, Zeiss).

### *G. mellonella* Infection Model

#### *G. mellonella* Survival Assay

For the primary determinant of the *in vivo* combined effects of FLU and MINO, a *G. mellonella* survival assay was performed as previously described (Benaducci et al., 2016; Rossi et al., 2016). Three *C. neoformans* (CN526) concentrations (5 × 10^5^, 5 × 10^6^, and 5 × 10^7^ cells/larvae) were used to determine the ideal inoculum concentration able to kill the larvae within 7 days at 37°C, and survival was evaluated daily. This timing was considered appropriate for the model because it fits with the insect life cycle.

In brief, four groups of randomly chosen larvae (20 for each group) were infected with 10 μl of CN526 inoculum (5 × 10^6^ CFU/ml) using a 50-μl microsyringe to inject the *C. neoformans* into the last left proleg of the larvae. Another group (Blank group) was injected with 10 μl of sterile PBS instead of CN526 inoculum and used as a negative control. Three hours later, treatments simulating human doses were used: 5.2 mg/kg FLU for the FLU group, 6.3 mg/kg MINO for the MINO group, a combination of 5.2 mg/kg FLU and 6.3 mg/kg MINO for the MINO/FLU group, and PBS for the control group. Treatment was administered only once. The caterpillars were observed for survival every 24 h for 7 days. Larvae were considered dead when they displayed no reaction to being touched. Larvae were cleaned with an alcohol swab before injection and were placed in the dark at 37°C for the duration of the experiment.

#### Fungal Burden Analysis

To evaluate the effect of the FLU/MINO combination on the fungal burden of larvae infected with *C. neoformans* (CN526), the colony count method was applied described previously (Sangalli-Leite et al., 2016; Lu et al., 2018). Five groups were randomly collected during days 0–4 of the experiment. Every day, three larvae from each group were randomly selected, then sliced. The slices were mixed in PBS-ampicillin, and a homogenate of samples from each group was serially diluted 10-fold, after which the resulting dilutions were plated on SDA plates supplemented with 100 mg/ml ampicillin. Colony counts were performed after incubation for 72 h at 37°C.

#### Histological Analysis

Histological studies were performed as previously described to observe the effect of the FLU/MINO combination on the tissue of *G. mellonella* larvae infected with *C. neoformans* (CN526) (Sangalli-Leite et al., 2016; Lu et al., 2018). Three larvae from each group were taken at 3 days post-injection. The caterpillars were fixed in 10% formalin for 24 h, then dehydrated with increasing concentrations of ethanol, washed with xylol, and finally embedded in paraffin. They were then sectioned serially at a thickness of 5 μm and stained using Periodic acid-Schiff (PAS). The resulting images were analyzed using an optical microscope with a 400× objective.

### Statistical Analysis

All experiments were performed three times on different days. Graphs were created, and statistical analyses were performed with GraphPad Prisma 7 and SPSS Statistics V17.0. Survival curves were analyzed utilizing the log-rank (Mantel–Cox) test. Fungal burden was analyzed using a Student’s *t*-test. A *p*-value of < 0.05 was considered statistically significant.

## Results

### Minocycline/Fluconazole in Combination Had Synergistic Effects Against FLU-Resistant *C. neoformans*

The MICs of MINO and FLU were assessed against fourteen *C. neoformans* isolates, and the resulting data are shown in Table 1. There are no accepted clinical breakpoints defining FLU resistance in Cryptococcus isolates, so we used MIC breakpoints of ≥16 μg/ml to determine FLU resistance. Five *C. neoformans* isolates (including H99) were FLU-susceptible with FLU MICs ranging from 1 to 4 μg/ml, whereas the other nine *C. neoformans* strains were FLU-resistant, with MICs of >32 μg/ml. The MICs of MINO against the tested strains were all >128 μg/ml, demonstrating the minimal intrinsic antifungal activity of MINO against these isolates.

For assessing the interactions of MINO with FLU against *C. neoformans*, MINO was combined with FLU to treat the FLU-resistant isolates. Synergism was observed in some of the nine tested FLU-resistant isolates, with FICI values ranging from 0.09 to 0.38. In contrast, for the combination of MINO and FLU against the FLU-susceptible strains, the FICI values indicated indifference (i.e., neither synergism nor antagonism between FLU and MINO).

**FIGURE 1:**
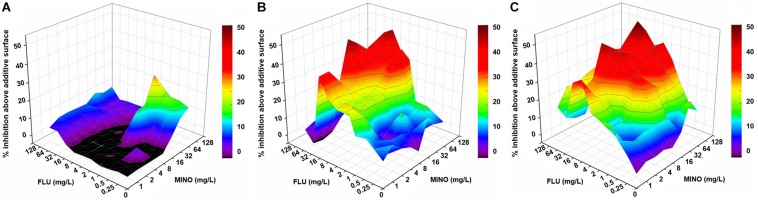
Three-dimensional model of MINO combined with FLU against *C. neoformans in vitro*. **(A–C)** Three-dimensional plots of MINO combined with FLU against the FLU-susceptible strain H99 **(A)** and the FLU-resistant strains CN18 **(B)** and CN526 **(C)** generated using the MATLAB program. The Δ*E* values are depicted on the *z*-axis; the peaks above the 0 plane indicate synergistic combinations, whereas the ridges below the 0 plane indicate antagonistic combinations. The color-coding on the right indicates that more effective drug combinations are shown in colors closer to the red at the top of the bar. FLU, fluconazole; MINO, minocycline.

Notably, when MINO was combined with FLU to treat FLU-resistant *C. neoformans*, doses of FLU ranging from 32 to 128 μg/ml significantly decreased the MINO MICs from >128 μg/ml down to 8–16 μg/ml, showing that MINO could dramatically increase the sensitivity of FLU-resistant *C. neoformans* to FLU. The FICI values were 0.09–0.38 following treatment with the combination of MINO and FLU; as these FICI values are substantially less than 0.5, they indicate strong synergistic effects between MINO and FLU against FLU-resistant *C. neoformans*. This synergism was further demonstrated by the Δ*E* model (Figure 1), with most of the Δ*E* values above the 0 plane. These observations indicate that MINO combined with FLU synergistically inhibited the growth of FLU-resistant *C. neoformans*. Thus, MINO might be a candidate for clinical use in combination with FLU against drug-resistant *C. neoformans*.

### Time-Kill Assay

**FIGURE 2:**
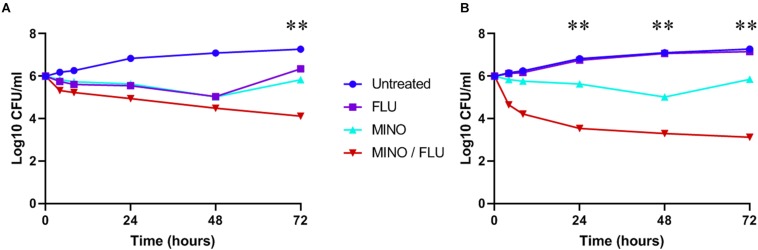
Time-kill analysis of *C. neoformans* strains H99 and CN526. **(A)** H99 (FLU-susceptible) was treated with 4 mg/L FLU, 128 mg/L MINO, or a FLU/MINO combination (2 mg/L FLU plus 64 mg/L MINO). **(B)** CN526 (FLU-resistant) was treated with 128 mg/L FLU, 128 mg/L MINO, or a FLU/MINO combination (4 mg/L FLU plus 8 mg/L MINO). The number of colony-forming units (CFU) remaining was quantified after various lengths of contact time (0, 4, 8, 24, 48, and 72 h). FLU, fluconazole; MINO, minocycline. The data presented are the means of three independent experiments. ^∗∗^*p* < 0.01, compared with the FLU-treated group.

Fungal viability was analyzed by CFU counting (time-killing curve). Treatment with FLU or MINO at their MICs reduced the cell viability of *C. neoformans* (H99 and CN526) but did not provide sustained killing over 48 h, despite the apparent susceptibility of H99 to FLU in the static assays (Figure 2). In contrast, the MINO/FLU combination displayed both rapid and sustained fungicidal activity over the course of the test for each strain. Moreover, there was a >2 log_10_ CFU/ml difference between CN526 and H99 in the viable counts of those treated with MINO/FLU at 72 h as well as a reduction of >3 log_10_ CFU/ml in comparison with the starting inoculum for CN526.

### Kinetics of Biofilm Formation

The metabolic activity of *C. neoformans* biofilms was determined using the colorimetric XTT reduction assay on polystyrene microdilution plates. The initial biofilm formation was observed following 2 h of incubation, at which point the *C. neoformans* were firmly adhered to the plastic surface. All *C. neoformans* samples produced biofilms and had a continuous increase in biofilm formation, regardless of their FLU susceptibility. The kinetics of biofilm formation in FLU-resistant strains trended higher than that in FLU-susceptible strains; however, the XTT reduction assay result showed no significant differences.

### MINO Synergized With FLU Against Different *C. neoformans* Biofilm Stages

All strains were found to be high biofilm producers (Supplementary Table S1). Three high biofilm-producing strains (CN18, CN117, CN526) and the H99 strain were used to test the interactions of MINO with FLU against pre-formed biofilms, and the results were interpreted by a FICI model as described above (Table 2). For the CN18, CN117, and CN526 biofilms that were pre-formed for ≤36 h, MINO decreased the sMIC_80_ of FLU from >256 to 8–64 μg/ml, with the FICI values ranging from 0.05 to 0.38. These FICI values are all substantially less than 0.5, indicating a strong synergism between MINO and FLU. For the biofilms that were pre-formed for >36 h, the sMIC_80_ of FLU when combined with MINO showed little difference compared with that of FLU alone, and the corresponding FICI values were all 2, indicating indifferent interactions between MINO and FLU. These data suggest that MINO can work synergistically with FLU against early stage biofilms but not against mature biofilms.

**TABLE 2:** *In vitro* interactions of MINO with FLU against *C. neoformans* biofilms.

Isolates^a^	Time(h)^b^	sMIC_80_ of drugs(μ g/ml)^c^				FICI^c^	IN^d^
		Alone		Combined			
		FLU	MINO	FLU	MINO		
H99	4	>256	>256	4	32	0.14	SYN
	8	>256	>256	8	32	0.16	SYN
	12	>256	>256	8	64	0.28	SYN
	24	>256	>256	16	64	0.31	SYN
	36	>256	>256	32	64	0.38	SYN
	48	>256	>256	256	256	2	ND
CN18	4	>256	>256	4	32	0.14	SYN
	8	>256	>256	8	32	0.16	SYN
	12	>256	>256	16	32	0.19	SYN
	24	>256	>256	16	32	0.19	SYN
	36	>256	>256	32	64	0.38	SYN
	48	>256	>256	256	256	2	ND
CN117	4	>256	>256	4	16	0.08	SYN
	8	>256	>256	4	16	0.08	SYN
	12	>256	>256	8	32	0.16	SYN
	24	>256	>256	16	32	0.19	SYN
	36	>256	>256	16	64	0.31	SYN
	48	>256	>256	256	256	2	ND
CN526	4	>256	>256	4	8	0.05	SYN
	8	>256	>256	4	16	0.08	SYN
	12	>256	>256	8	32	0.16	SYN
	24	>256	>256	16	32	0.19	SYN
	36	>256	>256	16	64	0.31	SYN
	48	>256	>256	256	256	2	ND

*^a^CN, Cryptococcus neoformans; ^b^Incubation period of pre-formed biofilms; ^c^sMIC_80_, minimum inhibitory concentration of drug that inhibited fungal growth by 80% compared with the growth control; FICI, fractional inhibitory concentration index; MIC_80_ values and FICIs are the median of three independent experiments; and ^d^IN, interpretation; SYN, synergism; ND, no difference.*

### Confocal Laser Scanning Microscopy (CLSM)

Confocal laser scanning microscopy was used to determine the viability of the cells adhered to a glass surface and to analyze the biofilm thickness. We applied two fluorescence dyes, Concanavalin A conjugated to Alexa Fluor 488 (CAAF) and FUN 1, to assess the cellular activity because they allow the observation of metabolically active structures in fungal cells. We tested MINO against biofilms formed by CN526 and evaluated the results using confocal microscopy. CAAF bound to the glucose and mannose moieties of the fungal cell wall (shown in the CLSM images as red staining), while the FUN 1 staining (shown in green in the CLSM images) was dense in the cytoplasm of metabolically active cell aggregates.

**FIGURE 3:**
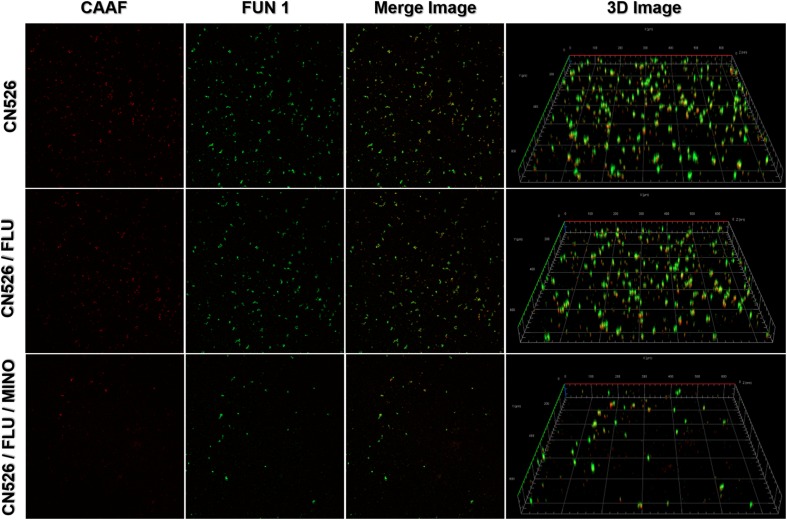
Confocal laser scanning microscopy (CLSM) of metabolically active cells in mature biofilms. Mature biofilms of CN526, produced as described above, were treated with FLU alone, FLU and MINO in combination, or left untreated. Staining with FUN1 fluorochrome (green) shows the cytoplasm of metabolically active cells. Alexa Fluor 488 (CAAF) staining (red) shows the presence of mannose in the cell wall. Representative images of CN625 following various treatments were captured and used to generate merged images as well as 3D images.

Images of biofilms formed by various strains were analyzed to determine their thickness and architecture. Sections of the three-dimensional images show that the biofilms in the MINO/FLU treatment group had a width of 2.1 μm, whereas those in the CN526 group had a thickness of 10 μm (Figure 3).

### MINO Protected *G. mellonella* Larvae Against *C. neoformans*

#### Survival Assay

**FIGURE 4:**
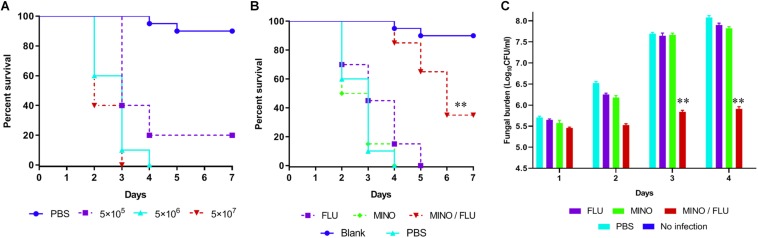
Survival rate and fungal burden of CN526-infected *G. mellonella* larvae treated with different drugs. **(A)** Different concentrations of CN526 (5 × 10^5^, 5 × 10^6^, and 5 × 10^7^ cells/larvae) were used to infect the *G. mellonella* larvae (20 larvae for each group) to determine the ideal inoculum concentration. The survival rates for each group were calculated. **(B)** CN526 (5 × 10^6^ cells/larvae) was used to infect the *G. mellonella* larvae (20 larvae for each group). Three hours after infection, the larvae were treated with 5.2 mg/kg FLU (FLU group), 6.3 mg/kg MINO (MINO group), or a combination of 5.2 mg/kg FLU and 6.3 mg/kg MINO (MINO/FLU group); the PBS group was used as a control. Treatment was administered only once. Another group (No infection group) that was injected with 10 μl of sterile PBS instead of CN526 was used as a negative control. The survival rate was calculated daily. **(C)** Experimental groups were established and treated as described in **(B)**. Every day, three larvae from each group were randomly selected and homogenized to determine the *C. neoformans* burden via inoculating dilutions of the homogenized caterpillars onto SDA solid medium plates, as described above. The data shown are the means of three independent experiments, and they were analyzed using a log-rank test. ^∗∗^*p* < 0.01, compared with the FLU-treated group.

In this study, *G. mellonella* larvae infected with CN526 were used to evaluate the *in vivo* interactions of drug combinations, primarily via the application of a survival assay. To determine an adequate ratio of CN526 to larvae for the virulence tests, survival curves were created for different amounts; a concentration of 5 × 10^6^ cells/larva was selected based on the results. The survival assay data show that 35% of the CN526-infected larvae in the MINO/FLU group survived the 7-day infection, whereas the survival rate of the CN526-infected larvae treated with FLU or MINO alone was 0%; thus, the survival rates of both the FLU and MINO alone groups were significantly different from that of the MINO/FLU group (*p* < 0.01). After 4 days of infection, the survival rates of the larvae were 15% for the FLU group, 0% for the MINO group, and 85% for the MINO/FLU group, indicating that FLU or MINO alone has weak or no effects on the infected larvae (Figure 4). The finding that MINO/FLU protected the larvae from CN526 infection and resulted in 35% of the larvae surviving 7 days of infection demonstrates the increased survival rates of larvae treated with the combination of MINO and FLU.

#### Fungal Burden Analysis

A fungal burden analysis was conducted to detect the effect of combined treatment with MINO and FLU on the fungal burden of *G. mellonella* larvae infected with CN526 (Figure 4). As expected, a gradual increase in fungal burden was observed in all groups. Encouragingly, the fungal burden of the MINO/FLU group was significantly lower than those of the untreated group and the drug monotherapy groups (*p* < 0.01), indicating that MINO/FLU significantly decreased the fungal burden of the CN526-infected larvae.

#### Histological Study

**FIGURE 5:**
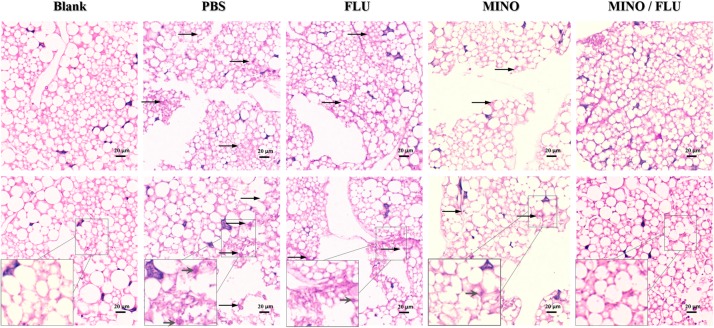
Histopathology of CN526-infected *G. mellonella* larvae treated with different drugs. CN526 (5 × 10^6^ cells/larva) was used to infect the *G. mellonella* larvae (20 larvae for each group). Three hours after infection, the larvae were treated with 5.2 mg/kg FLU (FLU group), 6.3 mg/kg MINO (MINO group), or a combination of 5.2 mg/kg FLU and 6.3 mg/kg MINO (MINO/FLU group); the PBS group was used as a control. Treatment was given only once. Another group (Blank group) that was injected with 10 μl of sterile PBS was used as a negative control; this group was not treated with CN526 or drugs. Three larvae from each group were randomly chosen after 3 days of incubation and cut into 5-μm sections. The sections were stained with PAS reagent and observed under a microscope. Arrows indicate *C. neoformans* cells were observed surrounded by hemocytes in inflammatory nodules and tissue destruction. Magnification, 400×.

A histological analysis was performed to characterize the *C. neoformans*-infected tissue of *G. mellonella* larvae after treatment with different drugs (Figure 5). The efficacy of the synergism was observed by histopathology of the caterpillars on day 3 post-infection. Severe tissue destruction and large quantities of *C. neoformans* cells were observed surrounded by hemocytes in inflammatory nodules in the PBS group that had been infected with *C. neoformans* compared with the blank (uninfected) group. Additionally, there were numerous *C. neoformans* cell nodules visible in the histology sections of the drug-monotherapy groups, in contrast with the much lower amount visible in the sections from the MINO/FLU group, suggesting that monotherapy with MINO or FLU has a limited effect against FLU-resistant *C. neoformans*. Notably, MINO/FLU could significantly reduce the number of FLU-resistant *C. neoformans* cell nodules in infected *G. mellonella* larvae, illustrating the high efficacy of MINO/FLU combination treatments.

## Discussion

Recently, the emergence of FLU-resistant *C. neoformans* has created great difficulties regarding the treatment of patients with *C. neoformans* infection (May et al., 2016; Recio and Perez-Ayala, 2018). To address this problem, researchers are searching for new antifungal agents or sensitizers of existing antifungal agents. Numerous antimicrobials or their analogs can enhance the efficacy of antifungal agents against *C. neoformans* (Sangalli-Leite et al., 2016; Donlin et al., 2017). MINO has been shown to have an inhibitory effect on *Staphylococcus aureus*, *Acinetobacter baumannii*, and *C. albicans* (Shi et al., 2010; Fragkou et al., 2019).

Biofilm formation by *C. neoformans* is related to its drug resistance (Jorjão et al., 2018). Furthermore, the drug resistance induced by biofilm formation hinders the treatment of *C. neoformans* infection. We hypothesized that treatment of FLU-resistant *C. neoformans* with a combination of FLU and MINO would have a synergistic effect via the inhibition of biofilm formation. To test this idea, we systematically evaluated the antifungal activity of MINO in isolation along with the interaction between MINO and FLU against *C. neoformans* both *in vitro* and *in vivo*. Our results yielded FICI values of 0.09–0.38, demonstrating that the combination of MINO/FLU has a synergistic effect on FLU-resistant *C. neoformans*. Additionally, our time-kill assay revealed that treatment with MINO/FLU produced rapid and sustained fungicidal activity.

Here, we found that MINO and FLU significantly cooperated against *C. neoformans* biofilms that had been pre-formed for ≤36 h, and the sMIC_80_ of FLU in combination with MINO was only 8–64 μg/ml, as compared with >256 μg/ml without MINO. With the extension of the biofilm preformation time, the biofilms became more mature, and the synergistic effect on these biofilms was weaker. CLSM images further support the finding that the MINO/FLU combination can inhibit early biofilm formation (<24 h) by FLU-resistant *C. neoformans*, but our CLSM data lacks brightfield images. Notably, indifference rather than synergism was observed for MINO/FLU on biofilms that had been pre-formed for >48 h, indicating that the combination of MINO and FLU may potentially be useful for the prevention or early treatment of biofilm-related diseases but is unlikely to be clinically useful in situations with mature biofilms. We speculate that the underlying mechanism of the MINO/FLU synergy may relate to restricting early biofilm formation.

*Galleria mellonella* is commonly used as a precursor model in which to study the virulence of pathogens and the efficacy of drugs before further testing is performed in mammalian models. Compared with mammalian hosts, this larvae infection model can quickly evaluate the effectiveness of drugs and the virulence of pathogens *in vivo*, and it has significant ethical and economic advantages (Benaducci et al., 2016; Jorjão et al., 2018). In the present study, we used a *G. mellonella* model to assess the combined effect of MINO/FLU on FLU-resistant *C. neoformans in vivo* and to determine the survival rate of larvae as a preliminary evaluation. After 4 days of infection, the larval survival rates of the FLU group, MINO group, and MINO/FLU group were 15, 0, and 85%, respectively, indicating that the protective effect of FLU or MINO alone on infected larvae was weak and that the combination of MINO/FLU could significantly improve the impact of these drugs on infection with FLU-resistant *C. neoformans*.

The results from our fungal burden analysis and histopathological studies also showed that a MINO/FLU combination protected larvae against FLU-resistant *C. neoformans* infection. Although the fungal load increased gradually in all groups, the fungal burden in the MINO/FLU group was much lower compared with that in all other infected groups. Histopathology was performed on the infected *G. mellonella* larvae to study the interaction of drug combinations *in vivo*. Compared with the Blank (uninfected) group, a large amount of tissue destruction and high number of yeast cell nodules were observed in the larvae infected with FLU-resistant *C. neoformans*. Additionally, fewer *C. neoformans* cell nodules were found in the MINO/FLU group compared with the control group and drug monotherapy groups. These results show that the combination of MINO/FLU could significantly lessen the damage to *G. mellonella* larvae caused by FLU-resistant *C. neoformans* at an experimental concentration. In conclusion, MINO can dramatically enhance the efficacy of FLU *in vivo*, which is consistent with the *in vitro* results described above.

Although preliminary evidence of effectiveness *in vivo* can be obtained by using invertebrate infection models, additional studies using mammalian infection models are required to confirm this finding. Additionally, minocycline may cause headaches, upset stomach, diarrhea, dizziness, unsteadiness, and mouth sores in people, and these side effects will need to be assessed in clinical studies. Furthermore, the mechanism of synergistic action in our observations remains incompletely understood, although, it has been reported that other antimicrobial agents with similar mechanisms, including tigecycline and rifampicin, also have anti-biofilm activity (Croes et al., 2010; Song et al., 2015). We speculate that the anti-biofilm activity of minocycline may be related to its inhibition of early protein synthesis. The relatively small number of isolates from a single geographic region that was studied here may limit the application of these results to other areas. Further investigations are planned to continue this research and address these questions.

In summary, the *in vitro* and *in vivo* findings reported in this paper suggest that the combination of MINO and FLU may be an effective treatment for FLU-resistant *C. neoformans* infections. This research provides an advance over recent work in the field by being the first report of synergistic effects against FLU-resistant *C. neoformans* by the combination of MINO with FLU. This study may provide new potential approaches for the treatment of fungal infections caused by other pathogens, such as *C. albicans* and *Pythium insidiosum.* Additional model system studies are needed before clinical trials can be commenced.

## Data Availability Statement

All datasets generated for this study are included in the article/Supplementary Material.

## Ethics Statement

This study was carried out in accordance with the recommendations of international ethical guidelines for biomedical research involving human subjects (CIOMS), and the protocol was approved by the Committee on Medical Ethics of The First Affiliated Hospital of Anhui Medical University. All subjects gave written informed consent in accordance with the Declaration of Helsinki.

## Author Contributions

QK and JL contributed to the conception and design of the study. QK and ZC organized the database. NL and HZ performed the statistical analysis. QK wrote the first draft of the manuscript. ZC, NL, HZ, YL, LH, and JL wrote sections of the manuscript. All authors contributed to manuscript revision and have read and approved the submitted version.

## Conflict of Interest

The authors declare that the research was conducted in the absence of any commercial or financial relationships that could be construed as a potential conflict of interest.

## Abbreviations

CN: *C. neoformans*
FLU: fluconazole
MINO: minocycline.
