# Supplementary material for: Minocycline and Fluconazole Have a Synergistic Effect Against Cryptococcus neoformans Both in vitro and in vivo
Source: Front Microbiol. 2020 May 5;11:836. doi: 10.3389/fmicb.2020.00836 (PMC7214679; doi:10.3389/fmicb.2020.00836)
Supplement: Supplementary file 1 [file Table_1.DOCX]

Supplementary Material

# Supplementary Tables

**Biofilm production**

The OD values obtained were used to calculate percent transmittance (%T) values. The %T value for every test sample was subtracted from the %T value of the blank to get the %Tbloc. The following interpretation scale was used: negative (%T < 5), + (%T = 5–20), + + (%T = 20–35), + + + (%T = 35–50), + + + + (%T > 50).

**Table S1 Biofilm production of all isolates used in this study.**

| Isolates | Mean% T  value ± SD | Biofilm Production |
| --- | --- | --- |
| CN1 | 68.16±6.98 | ++++ |
| CN2 | 45.38±8.07 | +++ |
| CN4 | 74.32±3.72 | ++++ |
| CN7 | 64.01±6.99 | +++ |
| H99 | 63.02±6.18 | +++ |
| CN18 | 60.27±5.02 | ++++ |
| CN26 | 57.2±6.68 | ++++ |
| CN45 | 36.73±7.84 | +++ |
| CN117 | 61.62±6.81 | ++++ |
| CN225 | 71.2±6.92 | ++++ |
| CN436 | 60.92±5.72 | +++ |
| CN526 | 73.78±6.66 | ++++ |
| CN593 | 35.68±4.11 | +++ |
| CN641 | 54.78±3.12 | ++++ |
